# Screening the Performance of a Reverse Osmosis Pilot-Scale Process That Treats Blended Feedwater Containing a Nanofiltration Concentrate and Brackish Groundwater

**DOI:** 10.3390/membranes14080164

**Published:** 2024-07-24

**Authors:** Christopher R. Hagglund, Steven J. Duranceau

**Affiliations:** Department of Civil, Environmental and Construction Engineering, University of Central Florida, 4000 Central Florida Blvd., Orlando, FL 32816-2450, USA

**Keywords:** nanofiltration, reverse osmosis, minimum liquid discharge, membrane concentrate, brackish groundwater, pilot-plant, iron sulfide, pyrite

## Abstract

A two-stage pilot plant study has been completed that evaluated the performance of a reverse osmosis (RO) membrane process for the treatment of feedwater that consisted of a blend of a nanofiltration (NF) concentrate and brackish groundwater. Membrane performance was assessed by monitoring the process operation, collecting water quality data, and documenting the blended feedwater’s impact on fouling due to microbiological or organic means, plugging, and scaling, or their combination. Fluorescence and biological activity reaction tests were used to identify the types of organics and microorganisms present in the blended feedwater. Additionally, scanning electron microscopy (SEM) and energy dispersive X-ray spectroscopy (EDS) were used to analyze suspended matter that collected on the surfaces of cartridge filters used in the pilot’s pretreatment system. SEM and EDS were also used to evaluate solids collected on the surfaces of 0.45 µm silver filter pads after filtering known volumes of NF concentrate and RO feedwater blends. Water quality analyses confirmed that the blended feedwater contained little to no dissolved oxygen, and a significant amount of particulate matter was absent from the blended feedwater as defined by silt density index and turbidity measurements. However, water quality results suggested that the presence of sulfate, sulfide, iron, anaerobic bacteria, and humic acid organics likely contributed to the formation of pyrite observed on some of the membrane surfaces autopsied at the conclusion of pilot operations. It was determined that first-stage membrane productivity was impacted by the location of cartridge filter pretreatment; however, second-stage productivity was maintained with no observed flux decline during the entire pilot operation’s timeline. Study results indicated that the operation of an RO process treating a blend of an NF concentrate and brackish groundwater could maintain a sustainable and productive operation that provided a practical minimum liquid discharge process operation for the NF concentrate, while the dilution of RO feedwater salinity would lower overall production costs.

## 1. Introduction

Nonporous pressure-driven membrane processes, such as nanofiltration (NF) and reverse osmosis (RO), produce high-quality product water commonly referred to as permeate and by-product water commonly referred to as concentrate [1,2,3,4]. NF and RO membranes are commonly used to remove dissolved contaminants from a solution via the solution diffusion mechanism [1,2,3]. The Donnan exclusion principle and hydrodynamic filtration are additional contamination removal mechanisms for NF membranes [5]. RO membranes are manufactured into a spiral wound (SW) thin-film composite (TFC) configurations typical for drinking water applications [6]. Six to eight SW–TFC configured membranes are commonly housed within a pressure vessel (PV) with a concentrate seal used to secure the structure [5]. Feed spacers are designed to separate membrane sheets, maintain a feed channel height, and create turbulence [5].

Typically, NF and RO processes are operated in a crossflow mode where a pressurized feed stream is pumped to the membrane surface, separating the water into two streams: permeate and concentrate [3,7]. The membranes are housed in PVs and arranged in multiple stages or passes with a decreasing number of elements to achieve a particular water flux set to minimize fouling [5]. In the treatment of brackish source water, the concentrate is fed to a subsequent set of PVs and hence the process is considered as having multiple stages. The quantity of the permeate that is produced from feedwater is defined as recovery. Typically, as the percentage of recovery increases, the amount of dissolved solutes in the concentrate increases and can exceed the solubility of sparingly soluble salts and result in scaling [8]. Recoveries for brackish water reverse osmosis (BWRO) processes are limited by sparingly soluble salts and commonly operate between 75 and 85% [8].

### 1.1. Fouling

Fouling is the process of accumulation, adsorption, or deposition of unwanted substances at the membrane surface which may lead to a reduction in permeate production and water flux, an increase in solute passage, downtime and maintenance, cleaning frequency, and overall cost [9]. Fouling may be reversible or irreversible and is divided into three categories for this review: (1) particulate, (2) scaling, and (3) biological and organic.

#### 1.1.1. Particulate Fouling

Particulate or colloidal fouling is caused by suspended particulates, including silt, clay, sand, precipitated crystals, silica, oxides metals, and organics or biological substances [10]. Colloidal fouling occurs when colloids are approximately equal or greater in size than the apparent membrane pore, causing blocking or cake layer formation, respectively [11]. In SW–TFC, while pore blocking may occur, particulate fouling is more of a concern due to concentration polarization (CP), which raises permeate solute concentrations and hinders permeate flux [11,12]. The common parameters used to assess colloidal fouling include turbidity, the silt density index (SDI), and the modified fouling index (MFI). It is recommended that the feedwater turbidity for an RO process remains under 1 NTU, although less than 0.3 NTU is preferred [5]. An SDI is used to assess the particulate fouling propensity of a feedwater by monitoring the time taken to filter a given volume of the stream through a 0.45 µm filter. An SDI value of less than 3 is recommended to minimize particulate fouling [5]. Cartridge filters (CFs), typically 1 or 5-µm, are used upstream of the nonporous process to minimize particulate fouling [5].

#### 1.1.2. Biofouling

Biological fouling, or biofouling, occurs when bacteria, algae, or other microorganism types form a biofilm on the membrane surface and secrete soluble organic matter known as extracellular polymeric substrates, polysaccharides, proteins, and humic acids [13,14]. The polysaccharide concentration in the water tends to increase with a decrease in pH and an increase in ionic strength [15]. Organic compounds, mainly natural organic matter (NOM), in the feedwater promote microorganism growth [11,16]. NOM can be classified into humic and fulvic acid categories, which are typically caused by plant, algae, or bacteria decay [17]. Note that NOM can be further divided into biodegradable and refractory subgroups, with humic and fulvic acids typically falling into the former category [17,18]. As the biofilm grows, viable bacteria and nonviable cells increase [19]. In this feedback loop, bacteria and nonviable cells increase the organics present, which then promotes biological growth. Feedwater total organic carbon (TOC) is a widely used measure for quantifying the amount of NOM in water and is commonly used to determine if organic fouling may occur. Feedwater containing TOC at a concentration less than 0.5 mg/L is unlikely to cause organic fouling, whereas it is likely to occur if the value is greater than 2 mg/L [13,20]. Moreover, an excitation emission matrix (EEM) can be used to track organic matter changes, particularly for humic and fulvic acids, aromatic proteins, and soluble microbials; fluorescence regional integration (FRI) divides excitation and emission ranges into sections that characterize the organics present in the sample [21,22].

#### 1.1.3. Scaling

Scaling occurs when inorganic salt ions agglomerate to form crystals that, when their equilibrium solubility limit is exceeded, precipitate onto (surface crystallization) or are transported (bulk crystallization) to the membrane surface or spacer material [23,24]. Moreover, it is important to consider the precipitation kinetics that dictates the speed at which scaling occurs [24,25]. Since inorganic salts accumulate near the membrane surface, CP may cause an increased scaling potential due to the amplified solute concentration on the active layer despite an increase in ionic strength occurring at the surface [6]. To minimize sparingly soluble salt precipitation, inhibitor chemicals are often added for pretreatment [6]. 

### 1.2. Concentrate Disposal and Minimum Liquid Discharge (MLD)

Concentrate disposal involves transporting residual streams to a location where human and environmental health are not affected [26]. Typical disposal methods for RO and NF concentrate are listed from most to least common: (1) surface water discharge, (2) sanitary sewer discharge, (3) deep well injection, (4) land application, and (5) evaporation ponds [27]. In Florida, the NF concentrate stream does not typically undergo additional treatment prior to or in lieu of disposal [26]. One alternative to the common disposal and treatment methods is MLD, in which waste discharge from water treatment plants is reduced [28]. In municipal drinking water applications, MLD processes are uncommon due to the large capital investment cost needed and their higher energy consumption [26].

Although a novel concept of minimizing NF concentrate disposal by incorporating the residual stream into the RO process feedwater would seem reasonable, little to no research has been published in this area. To illustrate this application, the beneficial reuse of the NF concentrate as a portion of RO feedwater was investigated at the bench-, pilot-, and full-scale levels [28,29,30,31,32]. In the bench-scale investigation, the NF concentrate was introduced into a treated sewage effluent stream upstream of an RO membrane, and it was found that the water flux across the flat-sheet increased likely due to the elevated organics in the feedwater [29]. In that work, Hafiz and colleagues assessed the foulants present on the membranes via scanning electron microscopy (SEM) and found the predominant atoms were carbon and oxygen, suggesting that organic fouling was present [29]. 

The City of Deerfield Beach (FL) pilot-tested an RO process treating blended feedwater containing an NF concentrate from a nanofiltration process treating surficial groundwater from the Biscayne Aquifer and brackish groundwater from the Floridan Aquifer (FA) [31,32]. The NF concentrate contained elevated concentrations of calcium, alkalinity, and silica, whereas the brackish groundwater had increased amounts of magnesium, sodium, potassium, sulfate, chloride, and TDS [32]. In addition, the authors monitored the normalized pressure drop and water mass transfer coefficient (K_w_) for the pilot-scale RO process treating a 50/50 blend of NF concentrate and brackish groundwater [32]. It was observed that the operational performance parameters monitored had declined by 20% relative to the initial conditions over a three-month period [32]. Although there appeared to be a potential for scaling without the need for inhibitor addition, this condition was not further investigated. While membrane fouling due to elevated organics in the NF concentrate was a concern, the pilot-scale study showcased that no significant organic fouling occurred [31,32]. The City of Deerfield Beach was able to demonstrate that an NF concentrate and brackish water blending application was possible [31,32].

### 1.3. Motivation for Further Study

Blended feedwater would allow for a practical MLD operation for NF processes and a reduced volume of brackish groundwater required for any brackish RO process. The objective of this work was to screen the concept of blending an NF concentrate with brackish groundwater upstream of an RO pilot-scale process. Operational performance and water quality could then be evaluated to assess fouling-related concerns caused by the blended feedwater. Blending an NF concentrate with brackish water source supplies could provide utilities with a means to achieve an MLD operation for the NF process while reducing energy costs for RO facilities due to feedwater salinity dilution.

## 2. Materials and Methods

### 2.1. Site Information

**Figure 1:**
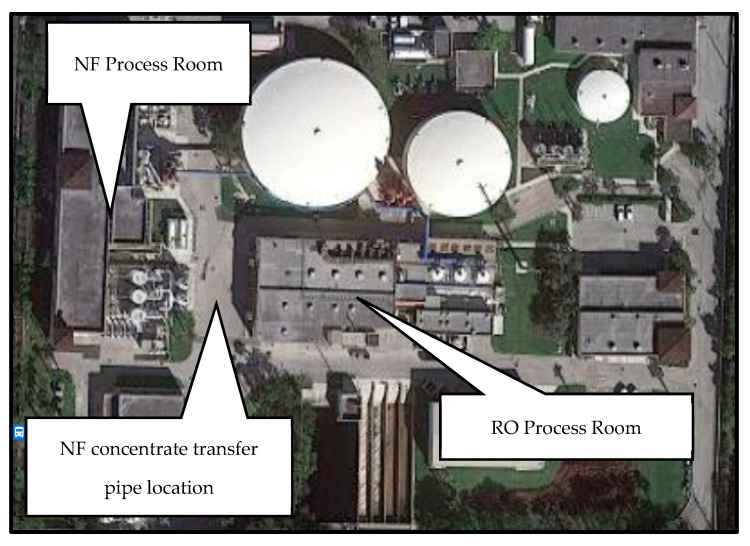
Aerial photograph of Jupiter Water Utility’s campus.

The study was conducted by the University of Central Florida’s (UCF) Water Quality Engineering Research Group (WQERG) at the Town of Jupiter’s Drinking Water Utility (Utility) (Jupiter, FL, USA) in partnership with Kimley-Horn and Associates (KHA) (West Palm Beach, FL, USA). UCF WQERG has conducted operations research including membrane performance studies, corrosion control evaluations, and distribution by-product assessments with the Utility and their consultants since 2009 [33,34,35]. The Utility serves more than 86,000 residents living in Jupiter, Juno Beach, and unincorporated areas of Palm Beach and Martin Counties. The Utility owns and operates two large-scale membrane processes co-located on the same plant site: (i) a 14.5 million gallon per day (MGD) (65,920 m^3^/day) NF membrane process treating surficial groundwater, and (ii) a 13.7 MGD (62,280 m^3^/day) RO membrane process treating brackish water. Figure 1 displays the locations of the NF and RO process rooms at the Utility.

### 2.2. NF Concentrate Supply

With respect to the NF facility, the Utility uses 45 surficial groundwater production wells capable of pumping approximately 21.8 MGD (99,100 m^3^/day) of water to the NF facility [33,36]. The surficial groundwater is blended upstream of its NF pretreatment processes that includes sand filtration, sulfuric acid, scale inhibitor addition, and cartridge filtration. The Utility’s NF process operates at an overall 85% recovery, with first and second stage operating at 67 and 47%, respectively. Currently, the Utility retails a mix of its NF concentrate water and sand filter backwash to the neighboring wastewater utility to blend with wastewater effluent and use for irrigation, per Florida Administrative Code 62-610.865 [37]. Approximately 90% of the blended stream is used for landscape irrigation [38]. The Utility is interested in an economically sound alternative to concentrate disposal by exploring MLD options, which includes the use of an NF concentrate for enhanced water supply management.

### 2.3. Brackish RO Well Supply

The Utility uses 11 Floridan Aquifer (FA) production wells capable of pumping approximately 14.7 MGD (66,830 m^3^/day) of water to the RO facility [36]. Since 2014, the Utility has rehabilitated FA wells to improve their performance and raw water capacity. However, chloride in the FA raw water has been observed to increase, likely due to saltwater intrusion [36]. The Utility’s brackish groundwater RO treatment system operates at a feedwater recovery of 75% [36].

### 2.4. RO Pilot Description

**Figure 2:**
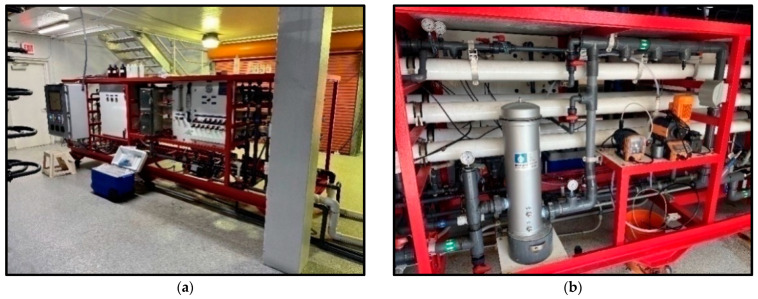
Jupiter Water Utility’s (**a**) RO pilot unit (**b**) and its pretreatment processes.

The Utility owns and operates a 22 gallon per minute (gpm) (0.0014 m^3^/s) RO pilot unit that has been used to test operational alterations prior to full-scale system implementation and is available for research purposes. The pilot unit comprises three 3-element and 4-element vessels to simulate 7-element vessels in the full-scale system. Note that the pilot-scale RO process operated at a feedwater recovery of 75%, which mimics the Utility’s full-scale process. This RO pilot process study was operated similarly to that in previous investigations that used solely brackish groundwater as the feed. Additionally, the options for industry-standard RO pretreatment including CF housing and scale inhibitor addition via an injection pump are available. The pilot skid contains a water quality sampling panel and a Supervisory Control and Data Acquisition (SCADA) control system. Figure 2 displays the RO pilot and pretreatment processes. 

**Figure 3:**
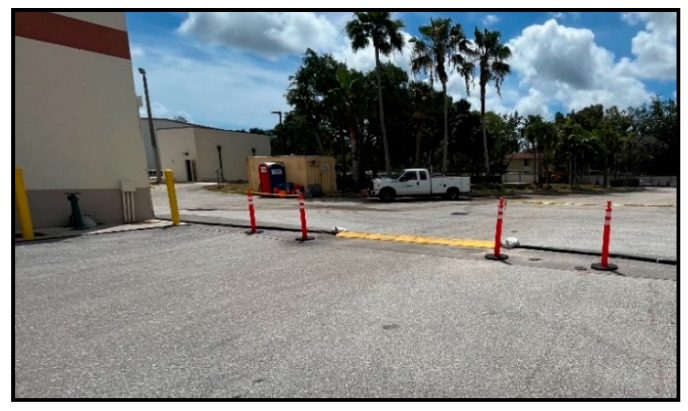
NF concentrate transfer pipeline between NF and RO process rooms.

The nanofiltration concentrate was transported from the NF process room to the RO process room via a pipe that ran approximately 100 feet between the buildings. Figure 3 shows an outside portion of the NF concentrate pipe. 

Table 1 shows the specifications of the membranes used in the first and second stages of the RO pilot-scale process in addition to the Utility’s full-scale NF process that produces the concentrate stream used in the blended feedwater. Note that the pilot-scale process relied on 4 inch-diameter, 40 inch-long membranes, and the full-scale facility consists of 8 inch-diameter, 40 inch-long elements.

### 2.5. Blend Ratio and Pretreatment Configuration

A bench-scale study was previously performed to understand the water chemistry of blended feedwater containing an NF concentrate and brackish groundwater at different blend ratios. After reviewing the water quality results, the Utility, their consultants, and UCF initiated pilot-testing of the feasibility of an NF concentrate and brackish groundwater blended feedwater; two pretreatment configurations were assessed.

The target blend ratio of the NF concentrate and brackish groundwater to prepare blended feedwater was based on the Utility’s full-scale operating conditions. Table 2 shows the target range for the percentage of NF concentrate integrated into the feedwater. The blended feedwater contained approximately 16.5% of the NF concentrate and 83.5% of pretreated brackish groundwater to bracket typical operating conditions; the blend ratio was maintained throughout the study. An analysis was performed to approximate the NF concentrate volume integrated into the full-scale RO blended feedwater stream. The capacity of each full-scale NF train (2013 gpm) and recovery (85%) were used to approximate the volume of nanofiltration concentrate produced each day. The RO pilot process remained at 75% recovery irrespective of the introduction of the NF concentrate into the feedwater. It was estimated that the volume of NF concentrate introduced into the RO feedwater to create the blend would range between 1.30 and 1.74 MGD, depending on full-scale plant operating conditions. The average of this range was assumed to equate to the volume of brackish groundwater reduced in a full-scale blending operation at this specific site. 

**Figure 4:**
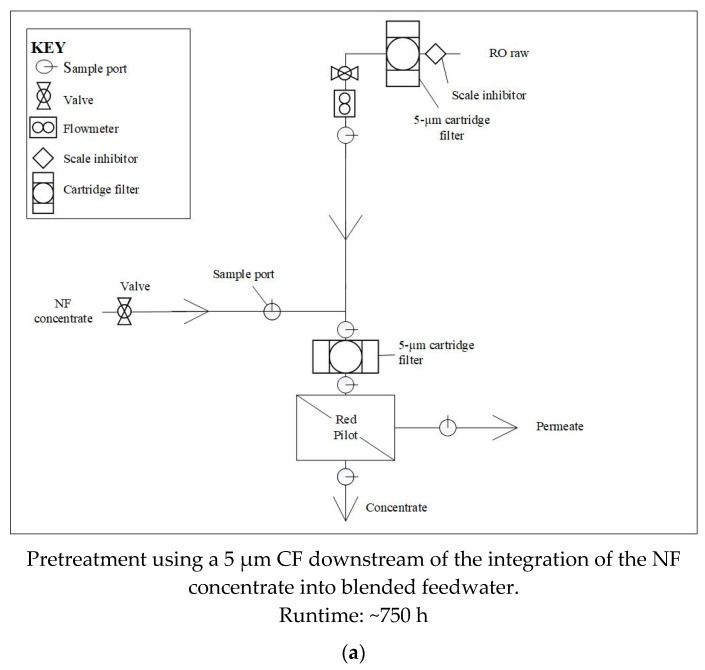

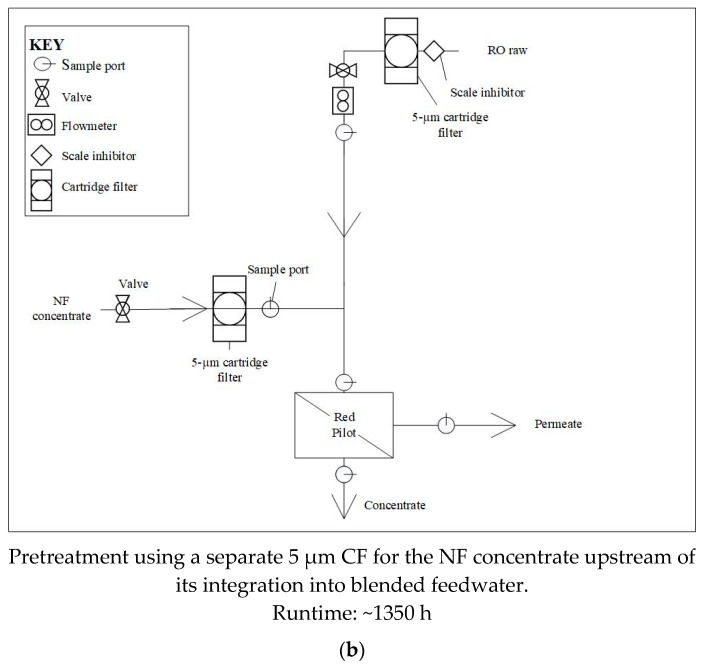
Pretreatment configurations for (**a**) Phase 1 and (**b**) Phase 2.

In the first pretreatment configuration tested (Phase 1), the NF concentrate was blended with pretreated brackish groundwater upstream of a 5 µm CF that fed the pilot unit. Note that the brackish groundwater pretreatment included a 5 µm CF and scale inhibitor addition. The performance of the RO membranes during Phase 1 was evaluated over approximately 750 h. The CF housing location was then moved to the NF concentrate line, upstream of its integration into the blended feedwater during subsequent testing (Phase 2). The RO membranes in Phase 2 were monitored for approximately 1350 runtime hours. Figure 4a displays the pretreatment configuration tested in Phase 1, and Figure 4b shows the location of the CF in Phase 2.

### 2.6. Water Quality and Operational Performance Analysis

Sample collection and water quality analyses were performed in accordance with Standard Methods for the Examination of Water and Wastewater (SM), the Environmental Protection Agency’s Test Methods for Drinking Water, and American Society for Testing and Materials (ASTM) [39,40,41]. Water quality was sampled from the feed, permeate, and concentrate streams via the pilot sample panel approximately once a week. The following water quality parameters were measured weekly: pH, temperature, conductivity, turbidity, TDS, oxidation reduction potential (ORP), alkalinity, dissolved organic carbon (DOC), chloride, bromide, sulfate, calcium, strontium, sodium, silica, barium, magnesium, iron, and potassium. In addition, several parameters were assessed to determine the propensity for the blend to cause fouling due to, but not limited by organic, microbiological, inorganic, or particulate means, as summarized in Table 3.

Operational data were typically collected thrice a day and analyzed in accordance with ASTM D4516-19A and D4472-08 [42,43]. The permeate, concentrate, and feed flows and pressures were recorded in addition to conductivity, which was measured for each PV. Note that in ASTM D4519-19A, the normalizing equations may vary depending on the membrane supplier; thus, the manufacturers’ software was also used in this study. Operational data were used to obtain the following membrane performance parameters: normalized permeate flow (NPF), pressure drop (ΔP), normalized salt rejection (NSP), water mass transfer coefficient, net driving pressure (NDP), and feed pressure (FP).

### 2.7. Feedwater Quality

Water quality was continuously monitored throughout the pilot study to assess membrane performance and identify parameters that could have contributed to fouling. Throughout the study, it was observed that the pretreated brackish groundwater contained higher concentrations of chloride, TDS, and sodium as well as lower amounts of sulfate and calcium than the NF concentrate stream did. Table 4 provides the average feed water quality collected during the screening study. 

**Figure 5:**
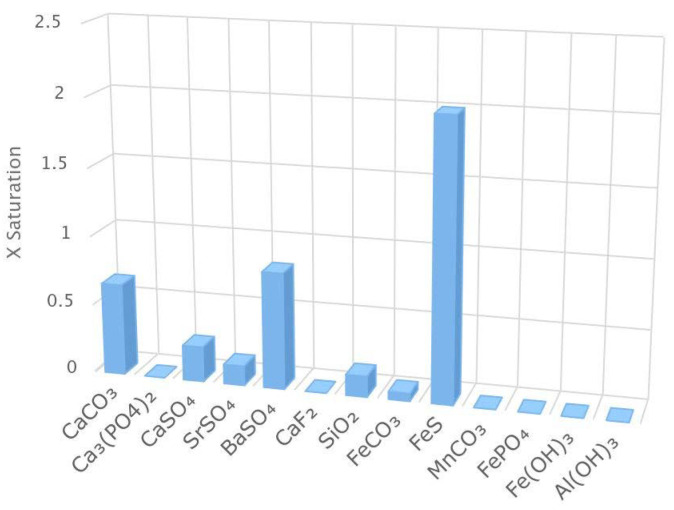
X saturation for the blended feedwater quality.

The feedwater’s precipitation potential was evaluated using Proton software (version 6.183.11) offered by American Water Chemicals (AWC) (1802 Corporate Center Ln, Plant City, FL 33563, USA). Using the Proton software, it was predicted that iron sulfide (FeS) had the highest saturation index (0.31) for the blended feedwater, as depicted in Figure 5, indicating a chemical dispersant be used. Additionally, calcium carbonate, calcium sulfate, and barium sulfate were observed to have a saturation index greater than zero in the scenario that scale inhibitor is not used. Note that the scale inhibitor (AWC^®^ A-111 Plus) is rejected in the Utility’s full-scale nanofiltration process, thus, the NF concentrate contains a concentrated portion of the antiscalant, approximately 6–8 mg/L. In addition, during pretreatment, the Utility adds a scale inhibitor (AWC^®^ A-102 Plus) to the RO feedwater. Therefore, the blended feedwater contains rejected antiscalant in the NF concentrate as well as a scale inhibitor introduced via an injection pump on the pilot skid. The scale inhibitors’ manufacturer, AWC, documented that the two chemicals are compatible and synergistic in this application [44]. 

### 2.8. Methods

#### 2.8.1. Analysis for Anions and Cations 

Samples were collected from the NF concentrate, brackish groundwater, blended feedwater, interstage, concentrate, and permeate streams to monitor the concentration of certain anions and cations present. Chloride, bromide, and sulfate in the streams were analyzed in accordance with SM 4110B using a Dionex ICS-1100 (Sunnyvale, CA, USA) ion chromatograph [45]. Barium, calcium, iron, magnesium, potassium, silica, sodium, and strontium were analyzed in accordance with SM 3120B using an Avio 200 Inductively Coupled Plasma Optical Emission Spectrometer (Waltham, MA, USA) [46].

#### 2.8.2. SDI

To assess particulate fouling, SDIs were performed on the blended feedwater and NF concentrate streams in Phase 2 at least once a day. The streams were filtered through a 0.45 µm disc hydrophilic membrane filters for 15 min [6,47]. Filtered water was collected in a 500 mL graduated cylinder and timed at the start and end of the test to calculate the SDI [6,47].

#### 2.8.3. Analysis for DOC 

DOC, a subset of TOC, was analyzed in this study in the NF concentrate, brackish groundwater, blended feedwater, interstage, concentrate, and permeate streams as it is typically more abundant than the particulate fraction [10]. In Phase 2, the method of standard addition was used, in which samples were highly diluted and spiked with a known concentration of the TOC standard. DOC was analyzed in accordance with SM 5310C using a Teledyne Tekmar TOC Fusion UV/Persulfate Analyzer (Mason, OH, USA) [48].

#### 2.8.4. Ultraviolet–Visible Spectrophotmetry (UV–VIS) 

Wavelength scans were performed to analyze absorbance in each sample at a one nanometer (nm) interval using a Hach DR6000 (Loveland, CO, USA). Wavelengths between 200 and 600 nm were assessed. 

#### 2.8.5. EEMs

**Figure 6:**
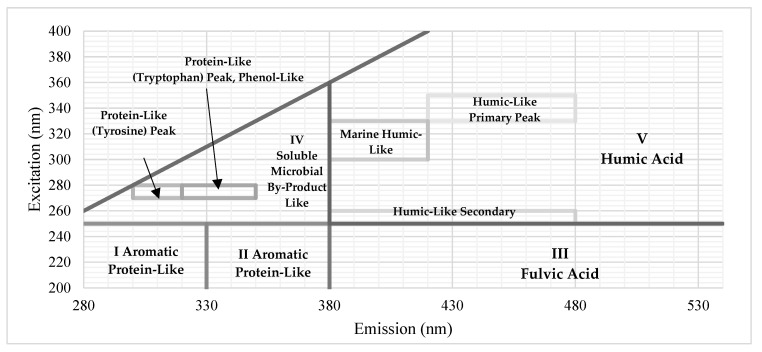
FRI region legend.

To characterize dissolved organic carbon components, a Shimadzu RF-6000 spectrofluorophotometer (Kyoto, Japan) was used on the NF concentrate, brackish groundwater, and blended feedwater streams. Samples were filtered with a 0.45 µm membrane filter to exclude large particulates from the analysis. Excitation wavelengths were measured every 5 nm from 200 to 400 nm, and emission wavelengths were measured every 1 nm from 280 to 600 nm. Additionally, a blank sample of deionized water was subtracted from the fluorescence spectra corresponding with each sample to minimize the Raleigh scattering effect [49]. The three-dimensional data were mapped as an EEM contour plot using OriginLab (Northampton, MA, USA). To identify DOM fractions corresponding with the EEM intensity peaks, a fluorescence regional integration (FRI) legend was used [22,49]. Figure 6 shows the FRI legend used in this study developed by Chen and coworkers [22]. 

#### 2.8.6. Biological Activity Reaction Test (BART)

BART was performed on the NF concentrate and blended feedwater streams towards the start of Phase 2 to quantify the amount of IRB, SRB, and SLYM present. At least 20 mL of sample was collected using Hach IRB, SRB, or SLYM test tubes. The samples were monitored for a minimum of eight days and routinely visually inspected for signs of bacterial reactions. Tables provided by Hach were used to quantify an approximate amount of bacteria present in the sample by matching it with the number of days until a reaction was observed [50].

#### 2.8.7. CF and Filter Pad Autopsy

The CF was sent to AWC for an autopsy, which involved the use of SEM and energy-dispersive spectroscopy (EDS) with superimposed elemental imagining^®^ (SEI) analysis. X-rays were produced as the sample material was bombarded with electrons from a Hitachi SU5000 SEM (Tokyo, Japan) and measured using a Bruker XFlash 6-60 dispersive spectrometer (Billerica, MA, USA) to display the elemental compositions of the foulants. Multiple sections (called spectrums) were selected at various magnifications to identify the atoms and concentrations present. Moreover, prismatic elemental delineation^®^ (PED) was used to display the locational presence of a given atom in a spectrum. Note that when reviewing the autopsy results, the atomic compositions were comparable for each spectrum, which indicated that the presented findings were representative of the entire CF.

**Figure 7:**
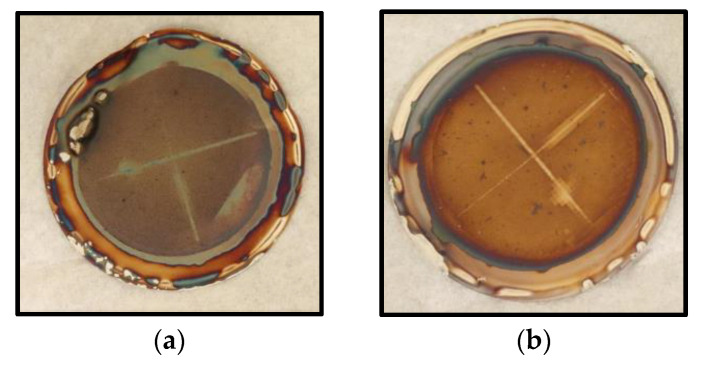
Silver SDI filter pad collected deposits corresponding with the (**a**) NF concentrate stream and (**b**) blended feedwater stream.

To supplement the CF autopsy findings, two 0.45 µm silver membrane filter pad analyses were conducted at the end of the screening study: (1) RO feedwater blend and (2) NF concentrate line. The silver membrane filter pads were selected to minimize carbon interference in the SEM and EDS analyses. It was expected that 20 L (L) of sample would flow through the silver filter pad; however, due to the low flow rate during the NF concentrate filter pad analysis, the bulk stream volume was reduced to eight liters. The silver filter pads are displayed in Figure 7a for the NF concentrate stream and Figure 7b for the blended feed.

## 3. Results and Discussion

The purpose of this section is to review and discuss findings from the RO pilot-scale study that investigated the treatment feasibility of blended feedwater containing an NF concentrate and pretreated brackish groundwater. This section focuses on the operational and water quality performance of the pilot RO process.

### 3.1. Operational Performance

**Figure 8:**
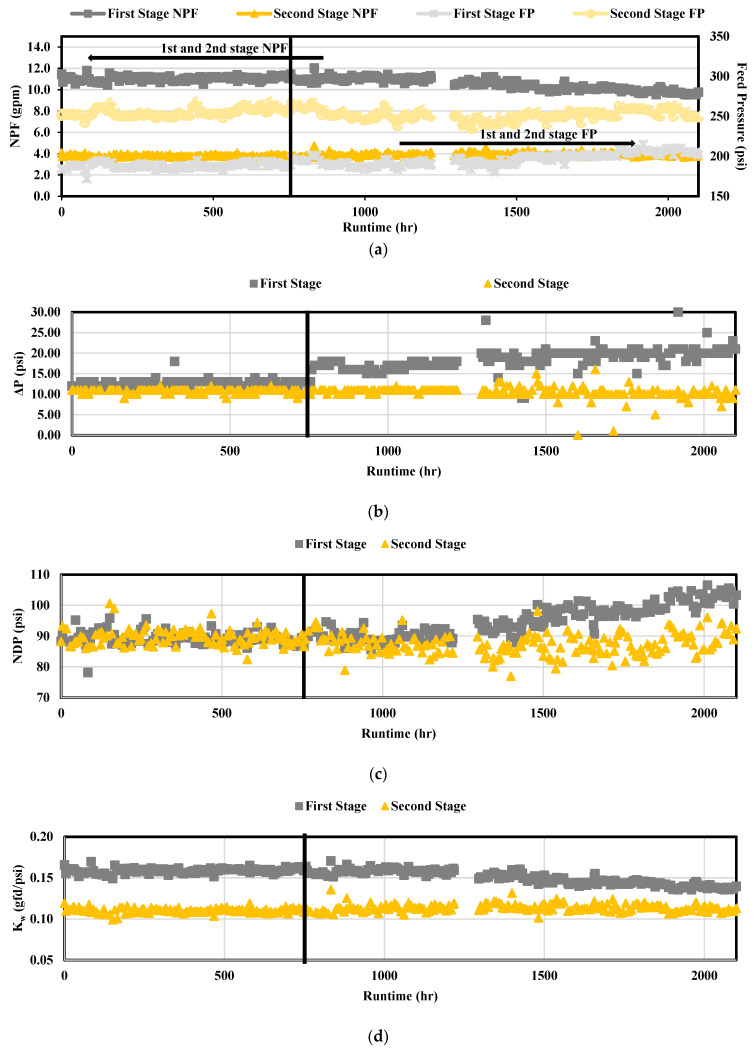
Operational performance including (**a**) NPF and FP, (**b**) ΔP, (**c**) NDP, and (**d**) K_w_ over approximately 2100 runtime hours.

Operational performance results are presented in Figure 8 for Phases 1 and 2. The FP and NPF were graphed together in Figure 8a, as the two parameters were typically proportional. It was found that the operational performance of the first stage was satisfactory as the FP and NPF appeared to remain at approximately 190 psi (13.1 bar) and 10.8 gpm (40.9 L per minute, lpm), respectively, over the 750 runtime hours in Phase 1. The second stage FP and NPF remained at approximately 254 psi (17.5 bar) and 3.70 gpm (14.0 lpm), respectively, for the duration of Phase 1. The first- (1.87%) and second-stage (0.720%) NDP percent difference was not observed to increase significantly, which suggested that fouling was minimal in Phase 1. For Phase 1, the membrane ΔP shown in Figure 8b remained at or near the average start up values of 13 psi (0.896 bar) and 11 psi (0.758 bar) for the first and second stage, respectively. The first-stage ΔP appeared to increase as the runtime increased; however, ΔP was not observed to increase in the second stage over the 750 runtime hours. 

In Phase 2, and particularly after 1300 total runtime hours, operational performance appeared to decline in the first stage. The second stage in Phase 2 operated similarly to Phase 1 with a minimal performance change. The first-stage NPF and FP values in Phase 2 ranged from approximately 9.78 to 11.27 gpm (37.0–42.7 lpm) and from 196 to 210 psi (13.5–14.5 bar), respectively. The most notable changes in operational performance for the first stage in Phase 2 were observed with NDP, displayed in Figure 8c, and ΔP, as the values ranged from 91.1 to 105 psi (6.28–7.24 bar) and from 18 to 21 psi (1.24–1.45 bar), respectively.

The increase in first-stage ΔP suggests that bacterial growth on the first stage membranes may have occurred over the study duration, particularly in Phase 2. Bacterial growth was documented to have occurred in the NF concentrate transfer pipeline; bacterial growth was not observed in the nanofiltration concentrate pipe located in the NF process room. Biological fouling is typically observed in the first stage of an RO process, often as a result of insoluble organic matter depositing on the membrane and releasing extracellular polymeric substances (EPS), which leads to a reduction in NPF and an increase in NDP [6,10,51]. Others have noted that the biofilm layer could increase CP yet at the same time hinder ion back diffusion, such that the membrane surface salt concentration increases, requiring an increase in the NDP [52].

Operational data also demonstrated that the membranes were capable of producing permeate without increasing pressures in Phase 1. Figure 8d illustrated that K_w_ remained at around 0.16 (0.066 lpm/bar) and 0.11 (0.045 lpm/bar) gfd/psi for the first and second stages, respectively. Due to the low relative difference between K_w_ in the first and second stages, operational performance appeared satisfactory in Phase 1. In Phase 2, K_w_ decreased after approximately 1300 total runtime hours due to the change in operating pressures required to produce permeate.

**Figure 9:**
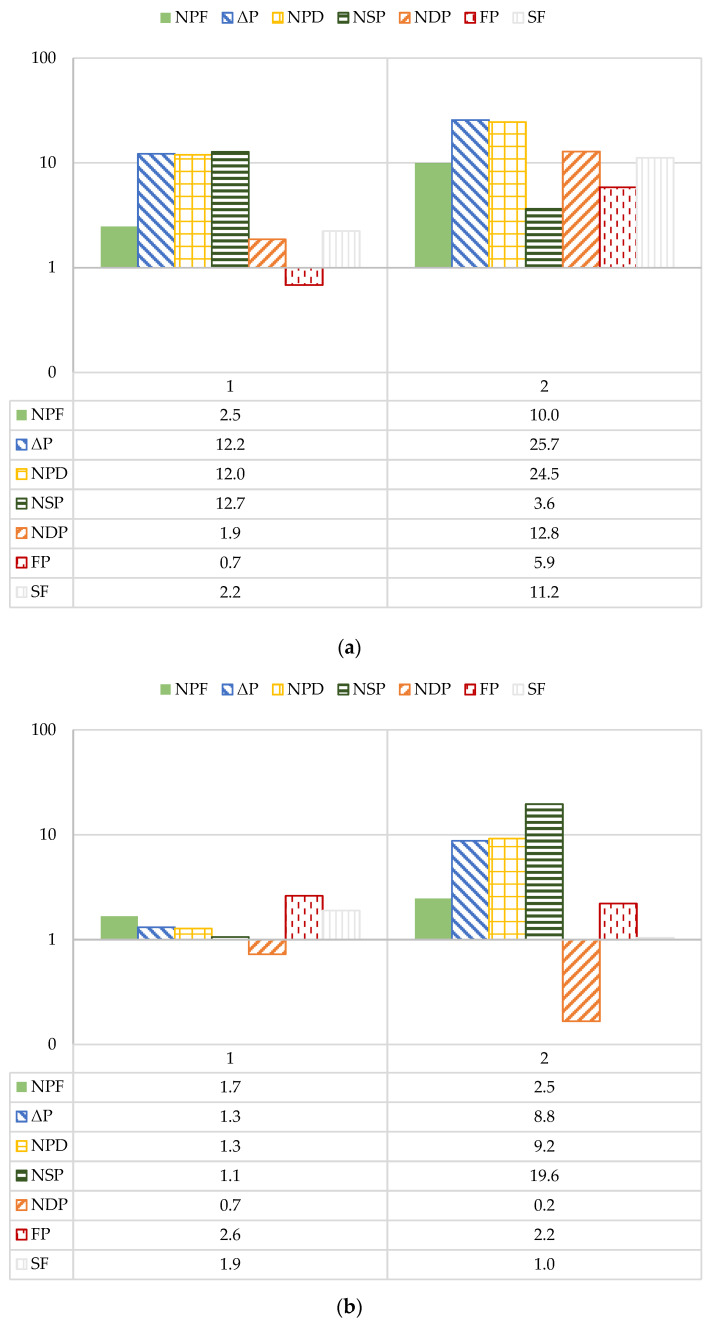
APD of operational performance parameters analyzed for the pilot’s (**a**) first stage and (**b**) second stage.

The absolute percent difference (APD) between the operational performance parameters during the first and last four days of the study was further assessed. The data are presented in logarithmic scale to easily determine the operational parameters that exceeded a 10% absolute change. In the first stage, ΔP and normalized pressure differential (NPD) were observed to exceed 10%, as shown in Figure 9a. ΔP and NPD are commonly monitored to establish a cleaning regimen; it is typically recommended that chemical cleaning is required when operational parameters exceed 10–15% relative to the start-up values [6]. However, additional literature suggests waiting until the NPD reaches between 15 and 50% [20,53]. Note that Van der Kooij and colleagues have used a relative percent difference to assess membrane performance, which is equivalent to the APD used in this study [51]. Thus, although the first-stage NPD APD exceeded 10%, it did not reach the 15–50% range that would require a chemical cleaning. Moreover, the NPD was the only analyzed operational parameter that consistently exceeded a percent difference of 10%, which started to occur at roughly 325 runtime hours for Phase 1 and within the first 50 h of Phase 2. Over Phase 1 and 2, the second-stage NPD sporadically exceeded 10% and 15%; however, the values were not observed to routinely exceed 10%. Similar findings have been observed in the study performed at the Deerfield Beach pilot [32]. The NPD and k_w_ of the RO pilot-scale process exceeded 20% in the first stage over the three study months [32].

In Phase 1, the calculated first-stage APDs for NDP and NPF did not exceed 10%. Second-stage NDP and NPF were not observed to exceed 5%. Thus, Phase 1 findings demonstrated that blending an NF concentrate into RO feedwater was achievable with a minimal performance decline resulting in MLD for the nanofiltration concentrate stream. However, in Phase 2, NPF and NDP exceeded 10% in the first stage; similar to the observations in Phase 1, the APD for NPF and NDP did not exceed 5% in the second stage. The findings showed that an operational performance decline occurred more significantly in the first stage in Phase 2 as displayed in Figure 9b. Since Phase 2 stage 1 NPF and NDP were observed to decline in performance, fouling due to organic, biological, or particulate means was suspected. The APD findings showed that a performance decline, mainly in Phase 2, occurred predominately in the first stage, which suggests fouling likely occurred due to bacteria present in the feedwater rather than from scaling, particulates, or organics. Biofouling was believed to have occurred in Phase 2 because the location of the cartridge filter was moved downstream on the NF concentrate line, which provided no pretreatment filtration of the blended feedwater. The reason that this change was made was to simulate the lowest-cost modification that would be required should full-scale implementation be performed at this specific site. The results suggested that additional engineering would be required to implement blending at the full-scale.

#### Pump Energy Consumption and Cost Comparison with and without NF Concentrate in RO Feedwater 

The approximate pump energy cost was calculated for the RO pilot process with and without the integration of an NF concentrate into the feedwater. Howe and colleagues documented a method to determine the specific energy consumption (E) and power (P), as shown in Equations (1) and (2), for a pump using the feed flow rate (Q_f_), pressure (P), pump efficiency (e), and permeate flow rate (Q_P_) [2]. In this study, the feed pressures corresponding with the first and second stages of the RO process were used. The cost was calculated based on a fixed rate of USD 0.129 per kilowatt-hour (kWh) [54].

(1) $$P_{w}=\frac{Q_{f}P}{e}$$

(2) $$E=\frac{P}{Q_{P}}$$

Table 5 displays the estimated pump energy consumption and corresponding cost for the RO pilot process with and without the integration of an NF concentrate into the feedwater. It was observed that the energy consumption increased by approximately 2% in the first stage of the RO pilot and decreased by 8% in the second stage. The decrease in pump energy consumption is attributed to the lower feed pressure when the NF concentrate is integrated into the RO pilot feedwater. Assuming the full-scale RO process is at capacity (13.7 MGD) and is operating similarly to the pilot unit, the integration of NF concentrate into the feedwater would approximately reduce the pump energy consumption by 10 kWh/MG, corresponding to a cost savings of approximately USD 1.30/MG (approximately USD 6500 per year for this facility). This cost savings does not include the reduced wellfield pumping energy required to transport the brackish feedwater to the plant. 

### 3.2. Water Quality

**Figure 10:**
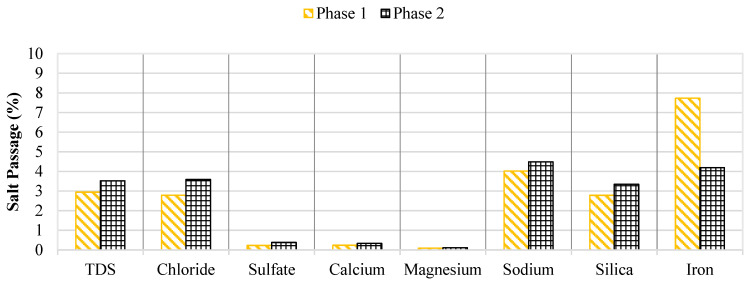
Percent passage of key water quality parameters in Phases 1 and 2.

Water quality was continuously monitored throughout the pilot study to assess membrane rejection and identify parameters that cause operational performance decline. Recall that brackish groundwater contained elevated levels of chloride, sodium, and TDS, whereas the NF concentrate had higher sulfate, calcium, and DOC contents. Membrane salt passage over the two study phases is provided in Figure 10.

**Figure 11:**
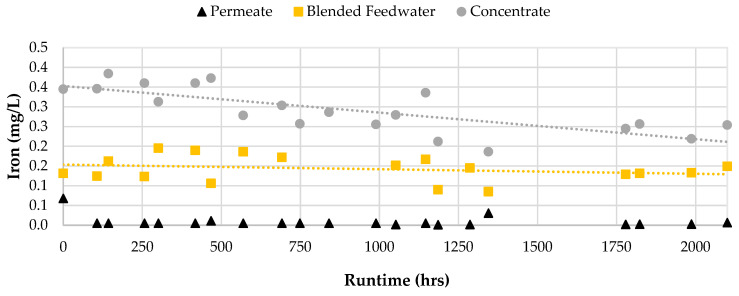
Iron concentration of the RO blended feedwater, permeate, and concentrate streams.

Generally, water quality did not vary over the 2100 runtime hours. However, iron passage appeared to decrease in Phase 2 as the feed concentration remained at approximately 0.15 mg/L. Initially, the iron concentration in the concentrate stream was around 0.36 mg/L, which was observed to decrease towards the end of the screening study. The decreasing trend in iron concentration for the concentrate stream suggested that the iron precipitated out of the solution and likely onto the membrane surface, which supports the decrease in operational performance findings in the first stage. Figure 11 displays the iron concentrations for the RO permeate, concentrate, and blended feedwater streams over the two study phases.

Recall that the manufacturer’s software identified FeS as the salt with the highest saturation index and most likely to precipitate out of solution (Figure 5). In aquatic systems, dissolved ferrous iron (Fe(II)) is typically formed under anaerobic conditions in which ferric iron (Fe(III)) oxides are reduced. Fe(II) is more common in aquatic groundwater, a closed system, such that Fe(III) would not be present unless an oxidant was introduced [55]. An anaerobic environment is created in groundwater systems when rainwater saturated with oxygen and organic matter percolates into an aquifer where bacteria oxidize the humic substances to reduce sulfate as shown in Equation (3) [10,55].

(3) $$2{\mathrm{CH}}_{2}O+{\mathrm{SO}}_{4}^{2-}↔2{\mathrm{HCO}}_{3}^{-}+H_{2}S$$

Sulfate-reducing bacteria have been observed to promote iron sulfide [56,57,58]. Iron sulfide, with a flake-like morphology, has been observed by others, thought to be due to the presence of sulfate-reducing bacteria and dissolved ferrous iron; the negatively charged cell walls of the bacteria offer a binding site for Fe(II) [58]. In addition, sulfate-reducing bacteria release sulfide that forms FeS with the iron present on the cell walls [58]. In aquatic systems, Fe(III) oxide-hydroxide and bisulfide are formed by sulfate and Fe(II) reactions, as displayed in Equation (4). Humic acid acts as an electron carrier between iron-reducing bacteria and iron oxide [59]. Additionally, the presence of Fe(II) and bisulfide may form iron sulfide, as shown in Equation (5). When combining Equations (4) and (5) and removing bisulfide, the result is shown in Equation (6). Note that FeS is typically less stable than pyrite (FeS_2_) but precipitates rapidly and is formed due to its fast kinetics [60].

(4) $$8{\mathrm{Fe}}^{2+}+{\mathrm{SO}}_{4}^{2-}+20H_{2}O↔8\mathrm{Fe}{\left(\mathrm{OH}\right)}_{3}+{\mathrm{HS}}^{-}+15H^{+}$$

(5) $$H^{+}+\mathrm{FeS}↔{\mathrm{Fe}}^{2+}+{\mathrm{HS}}^{-}$$

(6) $$9{\mathrm{Fe}}^{2+}+{\mathrm{SO}}_{4}^{2-}+20H_{2}O↔8\mathrm{Fe}{\left(\mathrm{OH}\right)}_{3}+\mathrm{FeS}+16H^{+}$$

Rickard and Luther claim that systems containing FeS are close to the FeS_2_ supersaturation limit that could cause the pyrite to nucleate by providing an active surface that enhances the nucleation process [55]. Pyrite formation is commonly initiated when Fe(III) oxide-hydroxide is reduced by bisulfide; this process is typically fast and is shown in Equation (7) [61]. FeS to FeS_2_ occurs via one of two pathways and requires an oxidant [58]. 

In the first pathway, FeS and elemental sulfur (S^0^) are oxidized via an external oxidant to form pyrite, which involves continuous sulfurization of iron sulfide and proceeds through a dissolution–precipitation pathway as shown in Equation (8) [62,63]. During this study, the CF was removed and visually inspected. It was observed that the CF was stained green, which suggested that green phototrophic sulfur-oxidizing bacteria (GSB) were present and aided in the initial formation of S^0^ and subsequent emergence of pyrite. GSB use light energy and hydrogen sulfide (H_2_S) as an electron donor to reduce CO_2_ and oxidize sulfur to S^0^ [64]. Duverger and colleagues investigated pyrite formation caused by sulfate-reducing bacteria and ferric phosphate (FePO_4_) and found that the FeS_2_ formation pathway likely occurs due to the formation of zero-valent sulfur caused by the reduction of FePO_4_ [58]. Moreover, S^0^ typically accumulates in the cytoplasm of sulfur-oxidizing or reducing microorganisms [65]. The operational performance decline in the first stage suggested that biological activity was present, which supports the claim that pyrite formation occurred as GSB was suspected and iron(II) decreased over the duration of the screening study.

**Figure 12:**
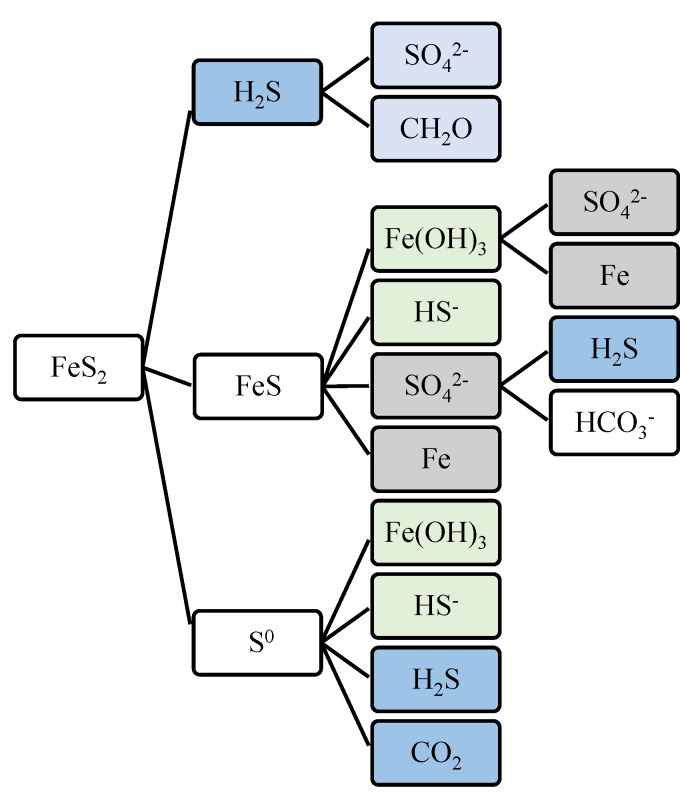
Pyrite formation pathways. Recurring parameters are shaded. SO_4_^2−^ and Fe are shaded gray, Fe(OH)_3_ and HS^−^ are shaded green, and H_2_S is shaded blue.

In the second pathway, FeS and H_2_S react to form pyrite and hydrogen as depicted in Equation (9). Interestingly, it has been documented that the presence of H_2_S promotes continued sulfate reduction [60]. During this research, sulfides were sporadically monitored over the study phases for the three feed streams. It was observed that the NF concentrate values were typically less than 0.5 mg/L S^2−^ compared to the relatively higher concentrations of 3.5 mg/L S^2−^ present in the brackish groundwater. The blended feedwater contained a similar concentration of sulfide to the brackish groundwater. Consequently, as has been documented by others, the formation of a FeS film on the membranes could have led to pyrite formation due to the presence of sulfide-reducing bacteria and dissolved ferrous ion [58]. Figure 12 illustrates the pyrite formation pathways, which include the contribution of sulfate, hydrogen sulfide, bicarbonate, and dissolved iron.

(7) $$12\mathrm{Fe}{\left(\mathrm{OH}\right)}_{3}+3{\mathrm{HS}}^{-}↔2\mathrm{FeS}+S^{0}+H_{2}O+3{\mathrm{OH}}^{-}$$

(8) $$\mathrm{FeS}+S^{0}↔{\mathrm{FeS}}_{2}$$

(9) $$\mathrm{FeS}+H_{2}S↔{\mathrm{FeS}}_{2}+H_{2}$$

**Figure 13:**
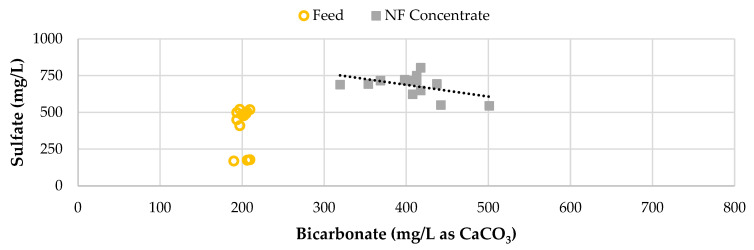
Sulfate concentration over 2100 runtime hours.

Figure 13 displays the blended feedwater’s and NF concentrate’s sulfate concentration and bicarbonate in the RO pilot streams over the duration of the study. The average sulfate passage across the study phases remained under 1% and close to 0.5%, with most permeate values below the laboratory detection limit of 2.0 mg/L. The sulfate concentration in the NF concentrate, RO concentrate, and blended feedwater appeared to decrease over time, with a more significant decline in Phase 2. It was found that as the bicarbonate increased, the sulfate concentrate decreased, suggesting that the sulfate was being reduced as expressed in Equation (3).

**Figure 14:**
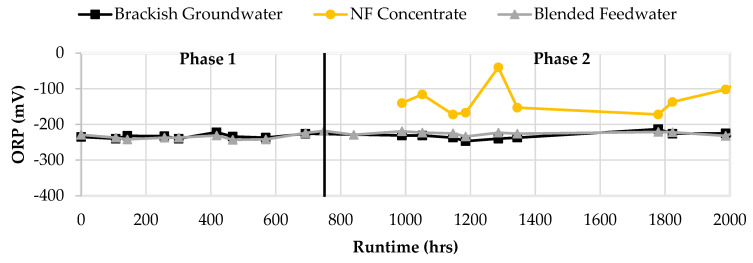
Brackish groundwater, NF concentrate, and blended feedwater ORP in Phases 1 and 2.

Figure 14 displays the ORP results in the feedwater streams. ORP for the brackish groundwater, NF concentrate, and blended feedwater streams were negative and typically less than −100 mV. The blended feedwater stream and the brackish groundwater typically had ORP values at −220 mV, which suggested that the water was anaerobic. The NF concentrate stream ORP appeared to fluctuate between −40 and −150 mV, which indicated that the stream may vary between anaerobic and anoxic conditions. 

#### 3.2.1. Particulate Fouling Observations

**Figure 15:**
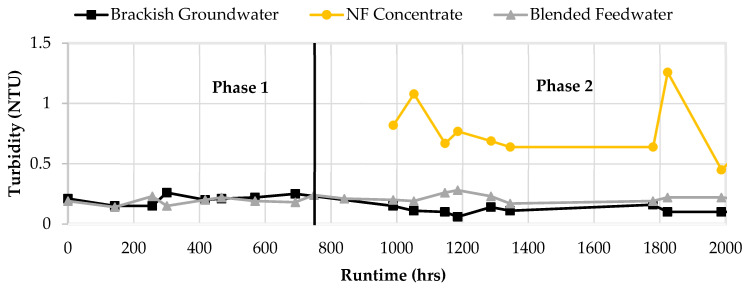
Turbidity for brackish groundwater, NF concentrate, and blended feedwater in Phases 1 and 2.

It was initially theorized that agglomeration may occur in the blended feedwater due to the water chemistry of the NF concentrate and brackish groundwater; an additional concern was raised because the NF concentrate was transported between the two process rooms such that agglomeration may have occurred in the transfer pipeline. Recall that turbidity values under 0.3 NTU are suggested to reduce the potential for membrane particulate fouling [6]. The average turbidity of the brackish groundwater and NF concentrate over the two phases were 0.13 and 0.65 NTU, respectively. It was shown that the NF concentrate stream increased the turbidity value of the feedwater; however, blend turbidity typically remained under 0.3 NTU due to the brackish groundwater diluting the steam. Figure 15 shows the turbidity results for Phases 1 and 2.

**Figure 16:**
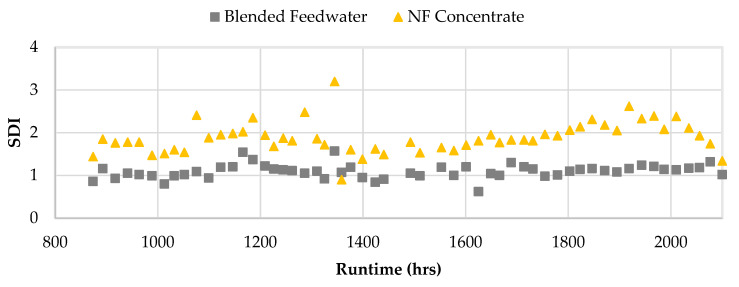
NF concentrate and blended feedwater SDIs from Phase 2.

Figure 16 shows the SDI values for the NF concentrate and blended feedwater streams starting in Phase 2. An SDI value less than 3 is recommended, and streams operating at or below that set point typically require no additional filtration pretreatment [6,53]. Interestingly, the SDIs for the NF concentrate also remained under 3 and were typically observed to be around 2, which suggested that the NF concentrate stream was unlikely to cause particulate fouling and likely does not require additional filtration pretreatment. Moreover, the CF ΔP was evaluated during Phases 1 and 2. The CF ΔP remained consistently under 2 psi, which suggested that particulate fouling was minimal.

#### 3.2.2. Biological and Organic Fouling Observations

**Figure 17:**
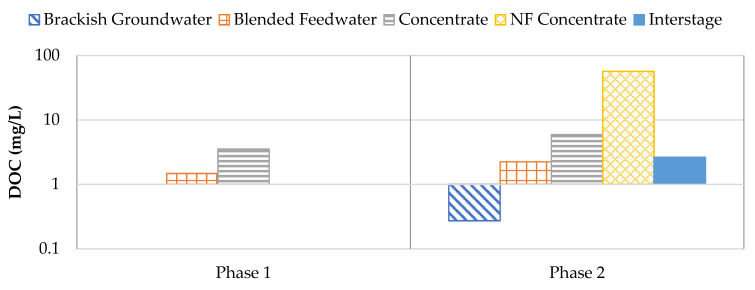
DOC results for the RO pilot process during Phases 1 and 2.

Figure 17 shows the DOC results for Phases 1 and 2. It was observed that brackish groundwater typically remained under the laboratory detection limit of 0.25 mg/L. The NF concentrate stream contained elevated levels of DOC, which typically ranged from 47.7 to 65.8 mg/L. As a result of the 16.5: 83.5 NF concentrate–RO brackish groundwater blend, the DOC in the blended feedwater stream typically ranged from 1.51 to 4.74 mg/L in Phase 2. It has been documented that DOC values exceeding 2 mg/L correspond to an increased likelihood of biofouling [20,53]. Additionally, UV-254 was analyzed to assess specific ultraviolet absorbance (SUVA) for Phase 1 and 2. UV–254 and SUVA are commonly used to assess the presence of organics in water samples as the parameters can be correlated to TOC, or in this case, DOC [66,67]. SUVA values above four correlate to the presence of organic matter in the form of aquatic humic acid, and values below two signify the existence of assimilable organic carbon [67]. The SUVA values were typically above 4 throughout the study, which indicated that the constituents were mostly aquatic humic matter.

**Figure 18:**
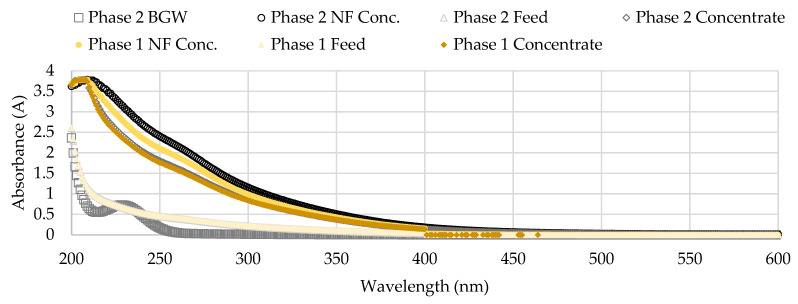
UV–VIS results of two sampling dates in Phase 2.

Figure 18 shows the UV–VIS results for single sampling events in Phase 1 (1 November 2022) and in Phase 2 (18 January 2023) for a wavelength range of 200 to 600 nanometers (nm). The NF concentrate had the highest UV values over the two sampling dates compared to those of the blended feedwater and brackish groundwater streams. The NF concentrate’s absorbance was observed to increase in Phase 2, which suggested that a higher concentration of organics was present. In addition, a slight peak was observed at around 260 nm for the assessed NF concentrate stream samples. Brackish groundwater (BGW) corresponded to the lowest UV values over the emission range; however, a peak was observed at around 230 nm for the sampling event in Phase 2. Trabelsi and others studied extracellular polysaccharide characteristics and found that UV absorption peaks at the 190–230 nm wavelength area correlate with amine, carboxyl, carbonyl, and ester functional groups, and 260–280 nm corresponds to aromatic and poly-aromatic compounds [68]. It has been documented that polysaccharides pose a threat to the operational performance of the membrane due to its influence on cake layer formation [10]. The suggestion that polysaccharides were present in the NF concentrate stream support the claim that biological fouling occurred in the first stage; as mentioned, biological fouling involves the deposition of insoluble organic matter which releases EPS, in this case polysaccharides [6,10,51]. Note that the sampling occurred in the RO process room, which indicates that biological activity, possibly GSB, was present in the NF concentrate transfer pipeline.

**Figure 19:**
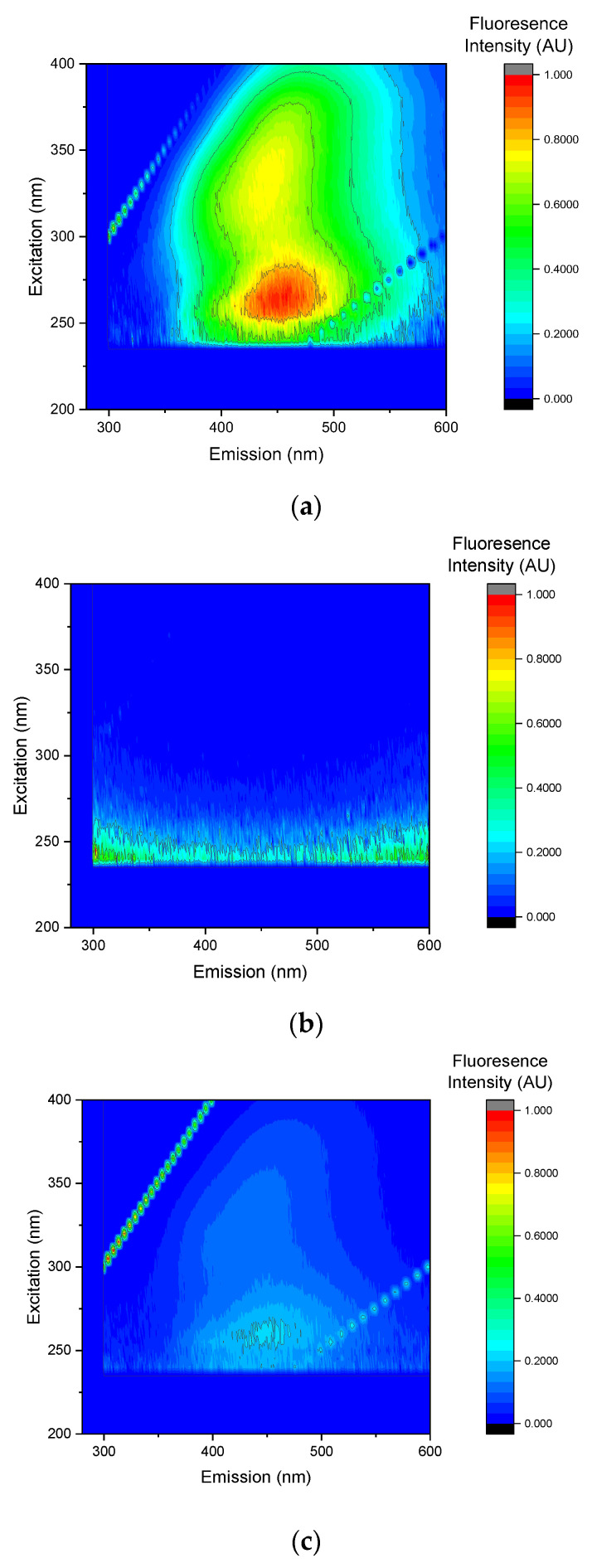
EEM results for the (**a**) NF concentrate, (**b**) brackish groundwater, and (**c**) blended feedwater.

In addition to SUVA and UV–VIS analysis, EEMs were used to characterize the DOC as shown in Figure 19. Note that in Phase 2, the NF concentrate was pretreated with a 5 µm CF and integrated into the pretreated brackish groundwater stream upstream of the pilot unit. The EEM findings support the SUVA results, as the organic matter present in the blended feedwater was predominantly humic acid. Moreover, the fluorescence results suggest that the NF concentrate contained the largest concentration of organic matter, similar to the blended feedwater findings, and the strongest intensity was observed in Region V, which corresponded to humic acid. Humic acid is often correlated with biofouling as it is oxidized to smaller organic compounds that can be assimilable for bacteria [10]. Additionally, humic substances can cause organic fouling via gel layer formation and adsorption [10,69].

**Figure 20:**
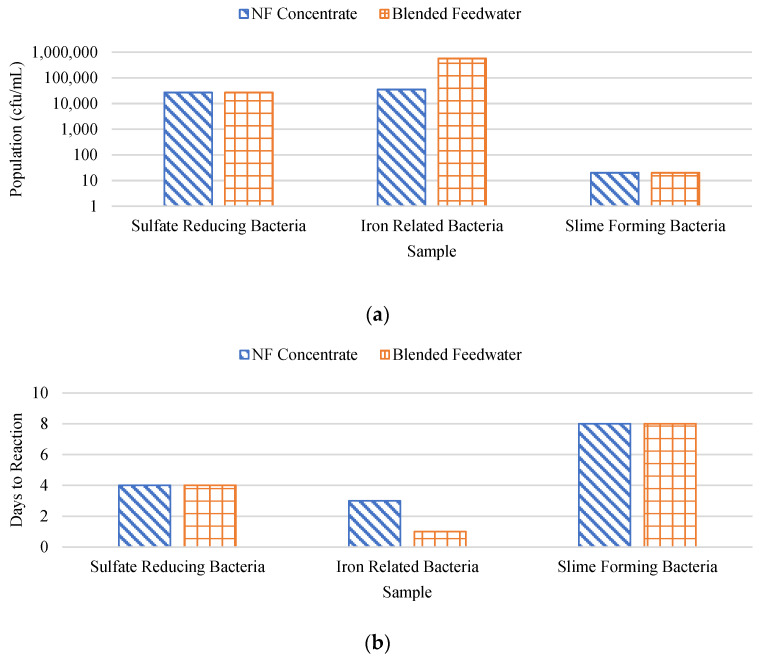
BART results for the NF concentrate and brackish groundwater streams in terms of (**a**) population (cfu/mL) and (**b**) reaction time (days).

Organics present in the NF concentrate stream likely promoted bacterial growth that resulted in the presence of secreted soluble organic matter, polysaccharides, and proteins [13,14]. The presence of these constituents in the NF concentrate and blended feedwater streams are corroborated by the UV–VIS and SUVA findings. Figure 20a,b shows the BART findings in terms of population and days until reaction, respectively. Note that the y-axis in Figure 20a is in logarithmic scale, and the units are in terms of colony-forming unit per milliliter (cfu/mL). It was found that the NF concentrate likely contained sulfate-reducing bacteria, dense slime bacteria, and anaerobic bacteria, while RO feedwater likely contained enteric bacteria and anaerobic bacteria. A study performed by Baker and Dudley found that fouled membranes typically consisted of between 10^2^ and 10^8^ viable bacteria (cfu/cm^2^) [70]. In this research, approximately 2.7 × 10^4^ cfu/cm^3^ sulfate-reducing bacteria were observed in blended feedwater and NF concentrate streams. Recall that sulfate-reducing bacteria oxidize humic acid to reduce sulfate to S^0^, which is a FeS and FeS_2_ formation pathway. Alternatively, GSB was suspected in the feed stream as the CF was stained green and may have oxidized H_2_S to S^0^, which promotes pyrite formation. In addition, the blended feedwater and NF concentrate streams contained approximately 5.7 × 10^5^ and 3.5 × 10^4^ cfu/cm^3^ iron-related bacteria, respectively, suggesting that biological activity aided in the formation of iron sulfide precipitants.

### 3.3. Autopsy Findings

Visual inspections of the CF after the conclusion of Phase 2 suggested that bacterial growth occurred, and it was believed that the NF concentrate stream provided favorable conditions for the microorganisms. Interestingly, biological growth was not observed in the NF concentrate pipe located inside the nanofiltration process room, which likely indicated that the growth occurred in the line between the RO and NF buildings. The findings are supported by a study where Chesters and colleagues observed an increase in bacterial growth at utilities that use glass fiber-reinforced plastic pipes [71]. In addition, visual inspection of the first stage lead and second stage tail elements at the conclusion of the screen study showed the membranes were stained black. Appelo and Postma observed that iron sulfide stains the sediment black, which suggests that FeS was present on the RO membranes in this screening study [60].

**Figure 21:**
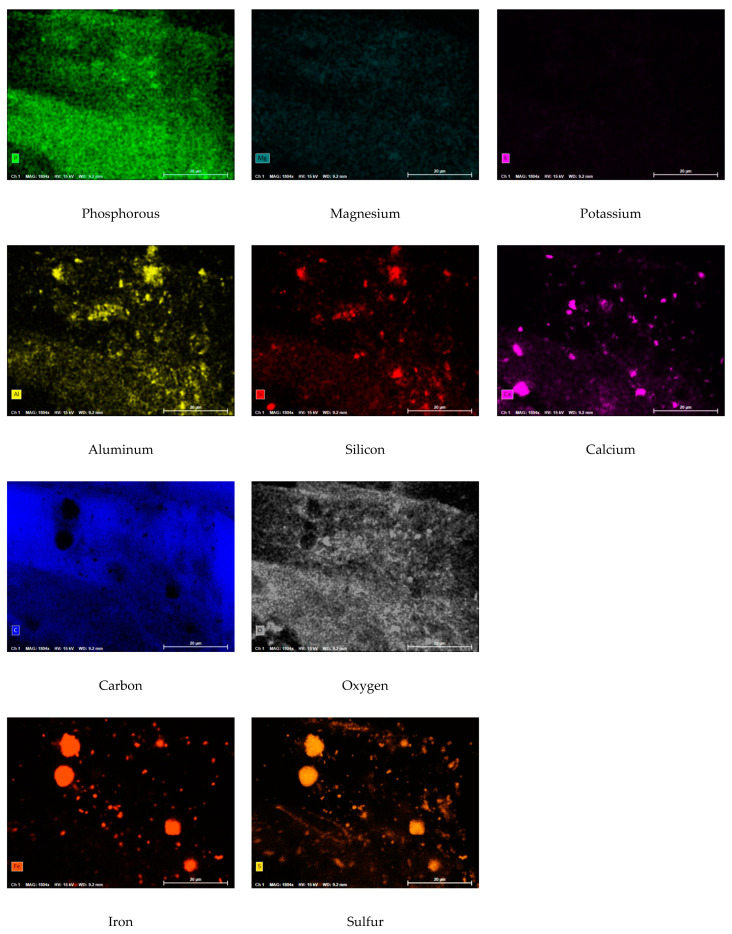
PED results for phosphorous (P), magnesium (Mg), potassium (K), aluminum (Al), silicon (Si), calcium (Ca), carbon (C), oxygen (O), iron (Fe), and sulfur (S).

**Figure 22:**
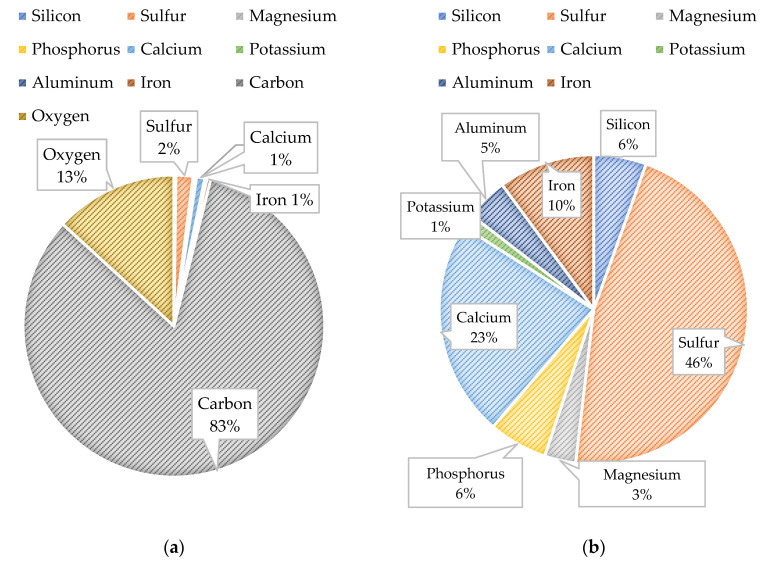
Composite pie chart findings for the CF (**a**) with carbon and oxygen and (**b**) without carbon and oxygen.

Figure 21 displays the PED results of the CF. Figure 22a,b shows the atomic percentages presented as pie charts with and without carbon and oxygen, respectively. The presence of organics on the CF was anticipated based on the feedwater quality findings. The autopsy results indicated that carbon (82.9%) and oxygen (13.2%) comprised the highest percentage of atoms present on the CF. It is important to note that the CF material (polypropylene) contains a backbone of carbon and hydrogen atoms; therefore, the atomic percentages may be impacted as the amount of carbon from the CF material or organics is indistinguishable. Additionally, iron and sulfur were identified on the CF at relatively low (<5%) concentrations.

Iron, sulfur, and biological matter were located at the same sites on the CF, which suggested that iron sulfide was present and formed due to microbiological activities. Iron sulfide was likely formed due to the water quality of the two feed streams and from sulfate-reducing bacteria identified in the feedwater. Moreover, it is theorized that the elevated concentration of sulfate in the NF concentrate stream was reduced to S^0^ due to the bacteria present in the transfer pipeline and supplemented by the elevated levels of DOC, in particular humic acid. The circular iron and sulfur shapes located on the PED were similarly observed by Duverger and others that documented pyrite spherules nucleating within an iron sulfide film after approximately one month of exposure [58]. In addition, the atomic percentage findings suggest that pyrite was predominant over FeS, which was speculated to have occurred due to (1) the presence of an external oxidant that continuously caused sulfurization or (2) from H_2_S.

The CF autopsy findings showed that organics, bacteria, iron, and sulfur were the predominant foulants present. Recall that operational performance was observed to decline at the start of Phase 2, whereas in Phase 1, the parameters did not significantly vary. The findings suggested that the blend of the NF concentrate with a larger concentration of organics and iron and brackish groundwater that contained elevated levels of sulfate promoted favorable conditions for the precipitation of FeS or FeS_2_, as well as organic and biological foulants. However, as the operational performance in Phase 1 did not significantly decline, it is theorized that the CF located downstream of the blend and upstream of the pilot was key to minimizing fouling for the RO membranes. In Phase 2, the first-stage RO membranes were observed to decline in operational performance and were stained black due to FeS or FeS_2_ formation.

**Figure 23:**
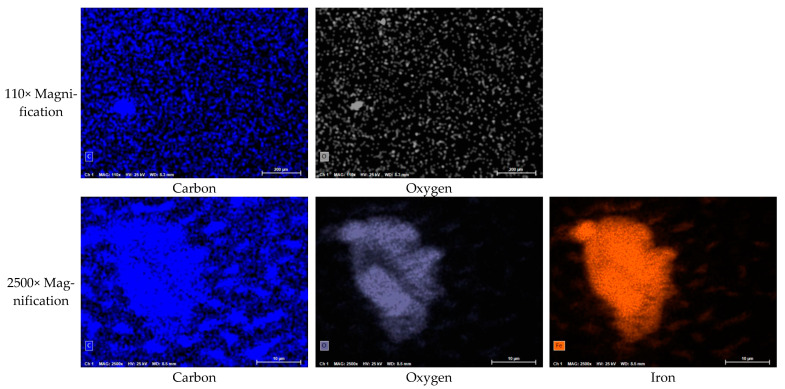
PED at 110× and 2500× magnification of the blended feedwater.

**Figure 24:**
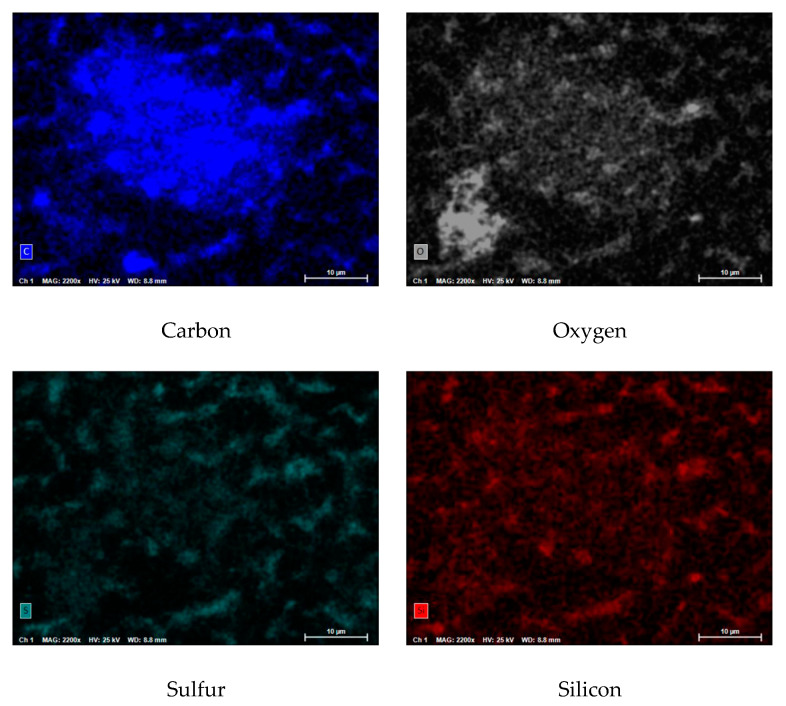
PED at 110× magnification of the NF concentrate for carbon (C), oxygen (O), sulfur (S), and silicon (Si).

**Figure 25:**
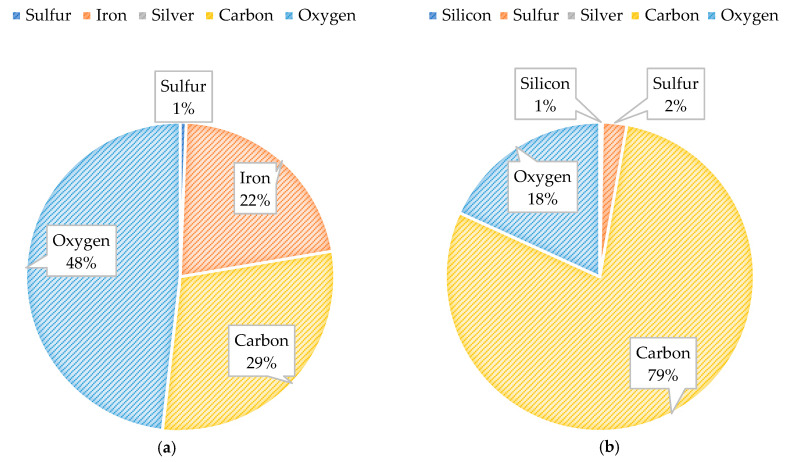
Composite pie chart findings of the (**a**) blended feedwater silver filter pad and the (**b**) NF concentrate silver filter pad.

Figure 23 displays the PED results at 110× and 2500× magnification for the SDI filter pad used to process the blended feedwater. Figure 24 shows the PED at 110× magnification for a spectrum from the NF concentrate SDI filter pad. Figure 25a,b illustrates the atomic composition of the blended feedwater and NF concentrate, respectively. The PED results for the blended feedwater showed that iron and oxygen were located in the same area, which indicated the presence of iron oxide. Initially, it was theorized that iron sulfide was present; however, PED showed that iron and sulfur were not located in the same regions. Recall that under anaerobic and sulfate-reducing conditions, iron oxide can be reduced and eventually form iron sulfide or pyrite. The NF concentrate silver pad autopsy analysis revealed that the predominant elements were carbon (79.0%) and oxygen (18.2%) suggesting that organics were present. Additionally, the blended feedwater pie charts indicated that carbon (29.5%) and oxygen (48.2%) were the predominant atoms present.

In addition, the water quality findings showed that blending the NF concentrate into the feedwater of an RO process was achievable and would result in MLD for the nanofiltration concentrate stream.

## 4. Conclusions

A pilot study investigating the RO treatment of blended feedwater containing an NF concentrate and brackish groundwater was completed. The findings indicated that bacteria and organics were present in the NF concentrate and blended feedwater streams via BART, fluorescence excitation–emission spectroscopy, and CF autopsy. The presence of sulfate, sulfide, iron, anaerobic bacteria, and humic acid likely aided in the formation of pyrite, which was observed on the position two element via autopsy analysis. Operational performance of the first stage appeared to decline in Phase 2 due to the presence of organics and bacteria not prevalent in Phase 1 operating conditions. Phase 1 and 2 operating performance differences could be explained by biogrowth-related fouling due to a change in CF housing location. This is supported by the APD findings, as the Phase 2 first-stage NPF and NDP values exceeded 10%, which was not observed in Phase 1. Water quality analyses confirmed that the blended feedwater contained little to no dissolved oxygen; additionally, SDI (<2) and turbidity (<0.3 NTU) results indicated the absence of a significant amount of particulate matter.

Initial performance results indicated that the operation of an RO process treating a blend of an NF concentrate and brackish groundwater could maintain a sustainable and productive operation under Phase 1 pretreatment conditions. Blending an NF concentrate with RO feedwater provides a practical MLD process operation for the nanofiltration process, while the dilution of RO feedwater salinity would lower overall operating costs. Pump energy consumption was observed to increase by 2% in the first stage and decrease by 8% in the second stage. However, the integration of the NF concentrate into RO feedwater was found to reduce feed pump energy consumption by 10 kWh/MG (or USD 1.30/MG). This study demonstrated the importance of the pretreatment filtration location for blended feedwater prior to an RO membrane process.

Water utilities that are reliant on using SW–TFC membranes to treat an analogous combination of surficial and brackish groundwater supplies may find the demonstrated success presented in this work applicable when considering a similar approach to minimize residual discharges to the environment. To be able to draw broader conclusions about the efficacy of blending an NF concentrate into RO feedwaters, analogous systems would likely have to evaluate their site-specific conditions themselves.

## Figures and Tables

**Table 1:** Membrane characteristics.

Parameter	Units	Full-Scale NF	Pilot-Scale RO
Membrane Type	-	Polypiperazine SW–TFC	Polyamide SW–TFC
Array	-	63:18	4:2
Membranes/PV	-	6	3
Feed Spacer	mil	34	28
Area	ft^2^ (m^2^)	400 (37)	85 (7.9)
Dimensions	in (mm)	40 × 7.9 × 1.125 (1016 × 201 × 29)	40 × 3.9 × 0.75 (1016 × 99 × 19)
Permeate Flow Rate	gpd (m^3^/d)	12,500 (47) *	2600 (9.8) **
Stabilized Salt Rejection	%	>97.0 *	>99.7 **

* Values based on the listed testing conditions: 2000 ppm MgSO_4_, 70 psi (4.8 bar), 77 °F (25 °C), and 15% recovery. ** Values based on the listed testing conditions: 2000 ppm NaCl, 150 psi (10.3 bar), 77 °F (25 °C), 15% recovery, and pH 8.

**Table 2:** Water production by minimum and maximum blend operating conditions.

Parameter	Unit	Minimum Operating Condition	Maximum Operating Condition
Full-scale RO trains typically in operation	-	5	6
Full-scale NF trains typically in operation	-	3	4
RO in blended full-scale permeate stream	%	46	44
NF in blended full-scale permeate stream	%	54	56
NF concentrate in blended feedwater	%	15.4	17.1
NF concentrate flow into RO feedwater	MGD	1.30	1.74

**Table 3:** Summary of additional foulant-related parameters analyzed.

Test	Foulant	Purpose	Sample
SDI	Particulate	Assessed particulate fouling concerns in Phase 2 feedwater streams.	NF concentrate and blended feedwater
Biological activity reaction test (BART)	Biological	Used to identify iron-related bacteria (IRB), sulfate-reducing bacteria (SRB), and slime-forming bacteria (SLYM) in Phase 2.	NF concentrate and blended feedwater
Excitation–Emission Matrix Fluorescence Spectroscopy (EEMs)	Organic	Characterized organics in Phase 2 feedwater streams.	NF concentrate, brackish groundwater, and blended feedwater
Specific ultraviolet absorbance (SUVA)	Organic	Supplemented EEMs findings in identifying the types of organics present in feedwater and concentrate streams.	NF concentrate, brackish groundwater, blended feedwater, and RO concentrate
SDI silver filter pad autopsy	Organic, scaling	Used to evaluate the atomic composition present on the filter pad upstream of Phase 2 feedwater streams.	NF concentrate and blended feedwater
CF Autopsy	Organic, scaling	Identified the atomic composition present on the CF upstream of the blended feedwater stream in Phase 1.	Blended feedwater

**Table 4:** Average water quality for feed streams.

Parameter	pH	Temp.	ORP	Turb.	Con.	TDS	Alk.	Ca^2+^	Mg^2+^	SiO_2_	Na^+^	Sr^2+^	Cl^−^	SO^2−^	Fe^2+^	DOC
Unit	s.u.	°C	mV	NTU	µS/cm	mg/L	mg/L	mg/L	mg/L	mg/L	mg/L	mg/L	mg/L	mg/L	mg/L	mg/L
Blended Feedwater	7.09	24.3	−219	0.24	8320	4670	198	207	166	14.4	1220	11.8	2290	368	0.147	<0.25
Brackish Groundwater	6.89	26.3	−231	0.13	8750	5090	162	147	158	14.0	1400	11.0	2870	448	0.082	2.43
NF Concentrate	6.86	25.2	−140	0.64	2210	2010	442	411	20.2	16.6	70.9	2.87	72.2	623	0.472	56.8

**Table 5:** RO pilot unit average operating conditions, approximate energy consumption, and corresponding cost.

Parameter	Stage	Average Flow Rate		Average Pressure	SEC	Cost
		Feed	Permeate	Feed		
Units	-	gpm (m^3^/h)	gpm (m^3^/h)	psi (bar)	kWh/gal (kWh/m^3^)	USD/gal (USD/m^3^)
Brackish groundwater feed	1st	19.9 (4.52)	11.0 (2.49)	184 (12.6)	7.71 (2040)	0.994 (263)
	2nd	8.82 (2.00)	3.84 (0.87)	273 (18.8)	12.0 (3170)	1.55 (409)
Phase 1 Blended Feed	1st	20.0 (4.54)	11.1 (2.5)	188 (13.0)	7.87 (2080)	1.02 (268)
	2nd	8.87 (2.01)	3.86 (0.88)	255 (17.6)	11.1 (2940)	1.43 (379)

## Data Availability

The data are contained within the article.
